# Insights into Deep-Sea Sediment Fungal Communities from the East Indian Ocean Using Targeted Environmental Sequencing Combined with Traditional Cultivation

**DOI:** 10.1371/journal.pone.0109118

**Published:** 2014-10-01

**Authors:** Xiao-yong Zhang, Gui-ling Tang, Xin-ya Xu, Xu-hua Nong, Shu-Hua Qi

**Affiliations:** Key Laboratory of Tropical Marine Bio-resources and Ecology/RNAM Center for Marine Microbiology/Guangdong Key Laboratory of Marine Material Medical, South China sea Institute of Oceanology, Chinese academy of sciences, Guangzhou, China; California Department of Public Health, United States of America

## Abstract

The fungal diversity in deep-sea environments has recently gained an increasing amount attention. Our knowledge and understanding of the true fungal diversity and the role it plays in deep-sea environments, however, is still limited. We investigated the fungal community structure in five sediments from a depth of ∼4000 m in the East India Ocean using a combination of targeted environmental sequencing and traditional cultivation. This approach resulted in the recovery of a total of 45 fungal operational taxonomic units (OTUs) and 20 culturable fungal phylotypes. This finding indicates that there is a great amount of fungal diversity in the deep-sea sediments collected in the East Indian Ocean. Three fungal OTUs and one culturable phylotype demonstrated high divergence (89%–97%) from the existing sequences in the GenBank. Moreover, 44.4% fungal OTUs and 30% culturable fungal phylotypes are new reports for deep-sea sediments. These results suggest that the deep-sea sediments from the East India Ocean can serve as habitats for new fungal communities compared with other deep-sea environments. In addition, different fungal community could be detected when using targeted environmental sequencing compared with traditional cultivation in this study, which suggests that a combination of targeted environmental sequencing and traditional cultivation will generate a more diverse fungal community in deep-sea environments than using either targeted environmental sequencing or traditional cultivation alone. This study is the first to report new insights into the fungal communities in deep-sea sediments from the East Indian Ocean, which increases our knowledge and understanding of the fungal diversity in deep-sea environments.

## Introduction

Although once thought to be an uninhabitable milieu owing to its extreme conditions, the deep-sea is now recognized as a home to rich and largely microbial communities [1]. Whitman et al. [2] reported that deep-sea-derived microbial communities, mainly composed of bacteria and archaea, accounted for a total cellular carbon content of approximately 3×10^17^ g. Besides bacteria and archaea [3]–[5], fungi in deep-sea environments have been extensively studied [6]–[8]. The isolation of deep-sea fungi was first reported approximately 50 years ago from the Atlantic Ocean at a depth of 4450 m [9]. Recently, an increasing number of fungal species were found in several deep-sea environments, e.g. sediments from Gulf of Mexico [1] and Mariana Trench at 11500 m depth [10], calcareous sediments [11], the Chagos Trench at a depth of 5500 m [12] and the Central Indian Basin at about 5000 m depth [8]. Orsi et al. [13] revealed that the deep-sea sediments are vast habitats for fungal life where cell may live on geologic timescale and these active fungi have an overlooked role in organic carbon turnover, which provide a direct evidence for active fungal metabolism in the deep-sea environments. Despite recent advances, the distribution and diversity of fungal communities in deep-sea environments are still largely unknown. With the recent development of more advanced instruments designed for sampling and researching life at greater depths, there has been more interest in evaluating the diversity and ecological role of fungi from deep-sea environments [14]–[20].

Traditionally, fungal diversity studies on deep-sea environmental samples have been based on cultivation techniques [21]–[24]. Fungi from deep-sea environments do not necessarily require extreme culture conditions, and many studies on deep-sea fungi describe using cultivation methods under standard laboratory conditions [21], [22]. Currently, more than 120 fungal species have been isolated from deep-sea environments [6], [18], [21]–[24]. Targeted environmental sequencing analyses, however, have indicated that cultivable fungi are only a small fraction of the total number of fungi inhabiting deep-sea environments [7]. The molecular phylogenetic analysis of clone libraries constructed from environmental samples has become the gold-standard in fungal diversity research, and this technique is thought to be able to detect a wider range of fungi that more accurately represent the investigated environment [25]. To date, several unknown novel phylotypes including DSF-group with the phylum Ascomycota [17], [26], KML11 clade and *Rozella* in the new described Cryptomycota [26], [27] and BCGI clade [17], have been discovered by molecular phylogenetic analysis.

This technique of molecular phylogenetic analysis, however, can be easily biased at many steps of the process, such as PCR primer selection and the DNA extraction method used [20]. Previous studies have shown that the 18S rRNA technique is a valuable tool for assessing the global diversity of eukaryotes [28], [29]. This approach, however, is limited because identification is often restricted to the genus or family level [30]. Buchan et al. [31] reported that the internal transcribed spacer (ITS) regions in fungal rDNA exhibit a high degree of polymorphism between species and are thought to be highly conserved within species. ITS regions can provide better taxonomic resolution than 18S rDNA sequences [16].

To identify the maximum amount of fungal species in the five deep-sea sediments collected from the East Indian Ocean and to find new sequences for phylogenetic studies, environmental gene libraries were constructed after amplifying the sediment DNA using an ITS rRNA gene primer set. Furthermore, the culturable fungi in these sediments were also isolated and identified by amplifying and sequencing the ITS rRNA gene.

## Materials and Methods

### Ethics statement

All the five sampling locations in this study were included in high seas. Permits for sediment sampling were provided by Ministry of Foreign Affairs of the People’s Republic of China. No specific permissions were required for these locations and the field studies did not involve endangered or protected species.

### Study site and sample collection

Five deep-sea sediment samples (A–E) were collected using a Remote Operated Vehicle (ROV) during the East Indian Ocean Open Cruise in March 2013 (Fig. S1 in File S1). The coordinates of Sample A–E were shown in Table 1. The collected sediment samples were mostly undisturbed and compact. The average length of the sediment cores collected from these locations was approximately 30 cm. Sub-cores of these samples were collected from a box corer using an alcohol-sterilized PVC cylinder that had a 5 cm inner diameter. Subsections of these samples were cut from the sediment sub-cores and immediately stored in sterile plastic bags to avoid any aerial contamination [18]. The bags were closed with rubber bands and transported to the laminar flow hood in the laboratory on board. A portion of sediment from the middle of each sub-sample that had not been in contact with the PVC cylinder wall was removed using an alcohol-flame-sterilized spatula and placed in a sterile vial for fungal isolation [21]. Fractions of these sediments were immediately stored at −20°C for direct DNA extraction after fungal isolation.

**Table 1:** The coordinates of Sample A–E.

Samples	Latitude	Longitude	Depth (m)
A	−2°57′N	95°19′E	4810
B	00°00′N	90°57′E	4532
C	00°30′N	82°03′E	4530
D	7°57′N	89°27′E	4614
E	10°00′N	84°33′E	4571

### Fungal isolation and identification

The cultivation and isolation methods for fungi from deep-sea environments do not differ fundamentally from the methods used for fungi from shallow marine environments. Physiological analyses have demonstrated that deep-sea-derived fungi are able to grow in deep-sea salinity and at low temperatures [32]. Three different methods were used for fungal isolation in this study, including the particle plating method [33], dilution plating method [21] and low temperature (10°C) incubation method. These fungal isolation methods are described in more detail in a recently published paper by Zhang et al. [18].

Fungal isolates were identified using a combination of morphology characteristics and the internal transcribed spacer (ITS) sequences. Total genomic DNA was extracted from all of the selected fungal strains using a method described by Lai et al. [16]. From the genomic DNA, nearly full-length ITS sequences were amplified by polymerase chain reaction with the primers ITS1 (5′-TCCGTAGGTGAACCTGCGG-3′) and ITS4 (5′-TCCTCCGCTTATTGATATGC-3′) [34]. These fungal ITS gene sequencing and identification methods are described in more detail in a previously published paper by Toledo-Hernandez et al. [35].

### DNA extraction, PCR amplification and clone library construction

Environmental DNA was isolated from two grams of sediment sample from each frozen subsection of the sediment cores using a soil DNA extraction kit (Omega Bio-Tek, Inc., Norcross, GA, USA) according to the manufacturer’s instructions and sterile techniques to avoid cross contamination. The DNA samples from the five sediments were amplified using the primers ITS1 and ITS4. The polymerase chain reaction mixture (20 µl) consisted of 2 µl 10× PCR buffer (500 mM KCl, 100 mM Tris-HCl, 15 mM 1% (*w*/*v*) MgCl_2_, and Triton X-100), 1.6 µl of 2.5 mM dNTP, 0.8 µl of each primer, 0.2 µl of 5 U Taq DNA polymerase (Takara Biotechnology Co., Ltd., Dalian, China), 13.6 µl of water, and 1.0 µl of template DNA (10–100 ng). PCR was conducted using an Eppendorf Mastercycler (Eppendorf German Co., Ltd., Hamburger, German) and the following program: denaturation at 95°C for 5 min, 25 cycles of 30 s at 95°C, 30 s at 55°C, and 90 s at 72°C, and a final extension at 72°C for 10 min. Reaction mixtures lacking template DNA were used as negative controls. Amplified products were gel-purified, ligated with pMD18-T easy vector (Takara Biotechnology Co., Ltd., Dalian, China) and transformed into *Escherichia coli* cells following the manufacturer’s instructions. Transformants were grown overnight at 37°C on Luria-Bertani agar containing 100 µg/ml ampicillin. The presence of insert was confirmed by PCR with M13 forward and reverse primers. One microliter of broth containing the clone was added to 25 µl of PCR reaction mixture. The PCR protocol included an initial hot start incubation (5 min at °C) followed by 34 cycles of denaturation at 94°C for 30 s, annealing at 55°C for 30 s and extension at 72°C for 1 min, and then a final extension at 72°C for 5 min [8]. Clones containing the positive insert were further processed for plasmid isolation and purification using the Millipore plasmid preparation Kit (Millipore, USA). Sequencing of the PCR products from the plasmids was conducted by Invitrogen (China). A total of five environmental gene libraries were constructed from the DNA samples from the five deep-sea sediments. Approximately 100 clones were screened from each library.

### Phylogenetic analyses

All of the vector sequences from the sequenced fungal clones were analyzed using the rRNA Database Project CHECK_CHIMERA program to detect and eliminate the potential chimeric sequences. Pairwise alignment of the sequences was conducted using Clustal W in the MEGA software version 5.0. Conserved motifs were identified, and the sequences were trimmed manually. Clones were grouped into operational taxonomic units (OTUs) using a sequence similarity cut-off value of 98% and the Mothur software version 1.32.1 [36]. Rarefaction curves for the number of observed OTUs were calculated with fungal assemblage at each dataset. A representative sequence from each OTU was queried against an NCBI-GenBank BLASTN search.

### Nucleotide sequence accession number

The ITS sequences for 20 culturable fungal isolate representatives and 45 uncultured fungal clone representatives obtained in this study were deposited in GenBank under accession numbers KJ173524–KJ17352 and KJ173554–KJ173590.

## Results

### Phylogeny of environmental fungal ITS-rDNA sequences

A total of 515 clones from five deep-sea sediment samples A–E (Fig. S1 in File S1) from the East Indian Ocean were sequenced. Of the resulting sequences, 445 sequences were found to be fungal, and a total of 45 operational taxonomic units (OTUs) (Table 2) were identified after clustering based on a 98% sequence identity criterion. The other 70 clones (∼13.6%) were eukaryotic or chimeric in nature and were excluded from this study. Rarefaction curves (Fig. S2 in File S1) were constructed for the ITS clone libraries from samples A–E. Rarefaction curves for three samples (B, C and D) demonstrated a plateau, which indicates that the number of sequences analyzed may sufficiently represent the fungal diversity in these samples. While the rarefaction curves of Sample A and E did not reach a plateau. It is likely that the fungal diversity of the two samples is higher than what was detected in this study.

**Table 2:** Phylogenetic affiliations of uncultured fungi obtained from deep-sea sediment samples A–E.

OTU	Closest identified relative			The number of clones				
no.	Taxon (Fungal phylum)	GenBank accession no.	Similarity %	A	B	C	D	E
*OTU-01*	*Phoma glomerata* (Ascomycota)	EU273521	100			2		5
**OTU-02**	*Cryptococcus curvatus* (Basidiomycota)	KF472136	99			57		
*OTU-03*	*Phoma herbarum* (Ascomycota)	KC311476	99					1
*OTU-04a*	*Rhizoscyphus ericae* (Ascomycota)	JQ711893	98	1				
**OTU-05a**	*Sporobolomyces lactosus* (Basidiomycota)	HQ914907	99					1
*OTU-06a*	*Trichoderma asperellum* (Ascomycota)	KC479819	100	1				
**OTU-07a**	*Trichosporon moniliiforme* (Basidiomycota)	AF444415	100					27
OTU-08a	Uncultured fungus clone (Ascomycota)	GU211938	100				27	
*OTU-09a*	*Xeromyces bisporus* (Ascomycota)	GU733338	99	9				
OTU-10a	Uncultured ascomycete (Ascomycota)	EU046087	99	1				
*OTU-11*	Uncultured *Geomyces* (Ascomycota)	JQ346989	99	1				
**OTU-12**	*Basidiomycete* sp. (Basidiomycota)	EU871524	99	2				
OTU-13	Uncultured soil fungus (Basidiomycota)	DQ420877	100				14	
**OTU-14**	*Cryptococcus fragicola* (Basidiomycota)	AB035588	99		5			
**OTU-15**	*Cryptococcus podzolicus* (Basidiomycota)	FN428930	99	1				
**OTU-16a**	*Guehomyces pullulans* (Basidiomycota)	AF444418	100			2		
**OTU-17**	*Basidiomycete* sp. (Basidiomycota)	EU871524	99			2		
**OTU-18**	*Rhodotorula slooffiae* (Basidiomycota)	AB566328	99		14			
**OTU-19**	*Sterigmatomyces halophilus* (Basidiomycota)	NR073302	100	37				
OTU-20a	Uncultured compost fungus (Basidiomycota)	DQ365334	99		5			
*OTU-21*	Uncultured *Mortierella* (Zygomycota)	JF831505	100	1				
*OTU-22*	Uncultured *Mortierella* (Zygomycota)	JF831503	99	25				
OTU-23a	Uncultured soil fungus (Ascomycota)	EU826926	99	2				
*OTU-24a*	*Alternaria alternata* (Ascomycota)	GQ916545	99					39
*OTU-25a*	*Cladosporium tenuissimum* (Ascomycota)	AJ300331	100					3
*OTU-26*	*Alternaria* sp. (Ascomycota)	KF888649	99					2
*OTU-27*	*Aspergillus penicillioides* (Ascomycota)	AY373862	97	2				
*OTU-28*	Uncultured fungus clone (Ascomycota)	HQ143117	99				32	
**OTU-29**	*Candida etchellsii* (Ascomycota)	JQ653271	99		24			
**OTU-30**	*Candida inconspicua* (Ascomycota)	AB179767	99			6		
**OTU-31**	*Candida sake* (Ascomycota)	AJ549822	99				2	
**OTU-32**	*Candida xylopsoci* (Ascomycota)	FM178339	100				2	
OTU-33a	*Cladophialophora chaetospira* (Ascomycota)	EU035404	93	2				
*OTU-34*	*Cladosporium cladosporioides* (Ascomycota)	GU932679	99					2
*OTU-35a*	*Cladosporium sphaerospermum* (Ascomycota)	GU017501	100	2				
OTU-36a	Uncultured soil fungus (Basidiomycota)	DQ420860	89	2		1		
*OTU-37a*	*Eurotium rubrum* (Ascomycota)	AY373891	99	1				
*OTU-38a*	*Fusarium solani* (Ascomycota)	JQ910159	100		5			
**OTU-39a**	*Galactomyces candidum* (Ascomycota)	JN974290	100		22	19		11
**OTU-40a**	*Dipodascus australiensis* (Ascomycota)	HQ115737	99		4			
*OTU-41*	*Geomyces pannorum* (Ascomycota)	DQ189228	99	1				
**OTU-42**	*Hortaea werneckii* (Ascomycota)	GQ334385	99					2
*OTU-43a*	*Hypocrea virens* (Ascomycota)	GU130297	100	1			12	
*OTU-44*	*Phoma sp.* (Ascomycota)	HQ630999	98			1		1
*OTU-45*	*Leptosphaeria* sp. (Ascomycota)	AB752252	99					1
Total				92	79	90	89	95

OTUs marked by a letter (a) are new reports for deep-sea environments. Bolded and italicized OTUs are affiliated with yeasts and filamentous fungi, respectively, and the remaining OTUs are affiliated with unidentified yeasts or filamentous fungi.

**Figure 1:**
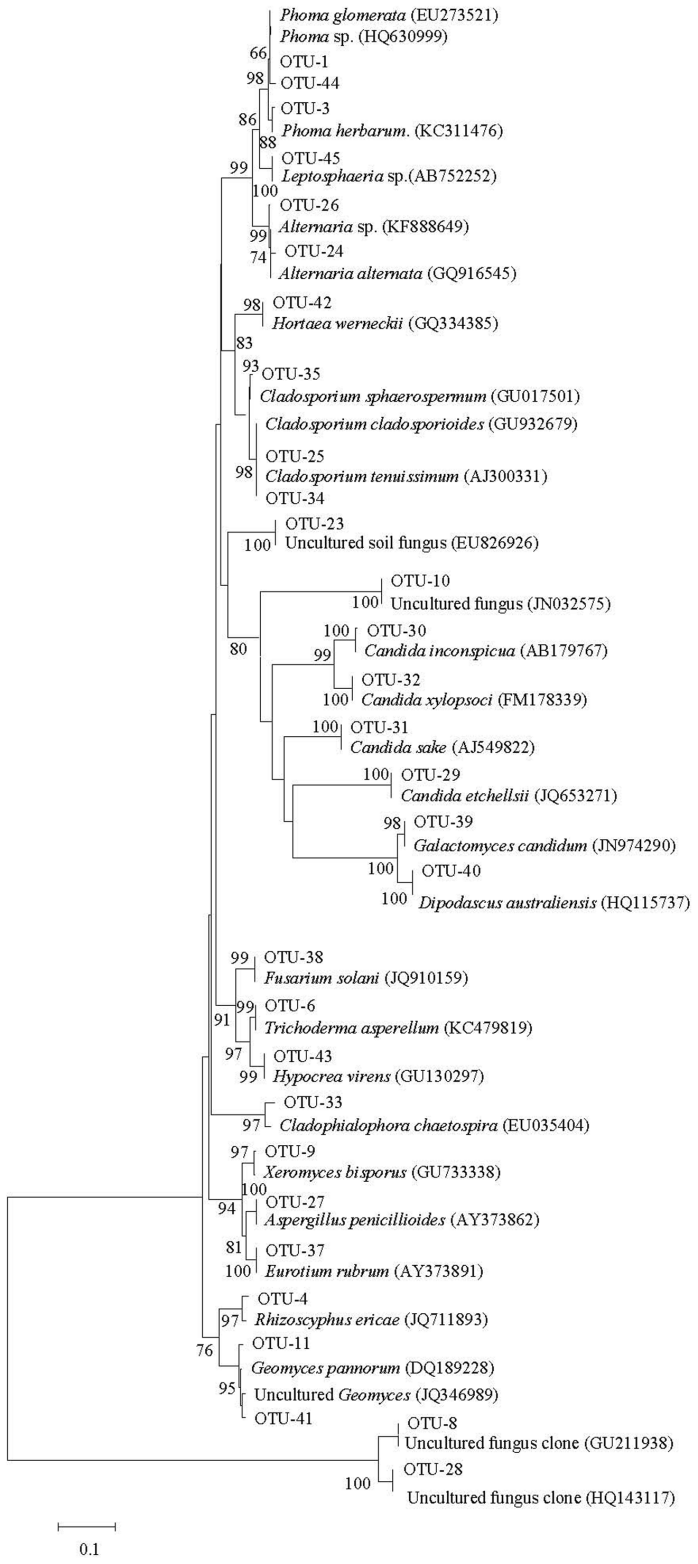
Neighbor-joining phylogenetic tree from analysis of ITS rDNA from 29 Ascomycota representative sequences in five libraries. The numbers at the nodes are the percentages indicating the level of bootstrap support based on a neighbor-joining analysis of 1000 resampled data sets. Only values >50% are shown. The scale bar represents 0.1 substitutions per nucleotide position.

**Figure 2:**
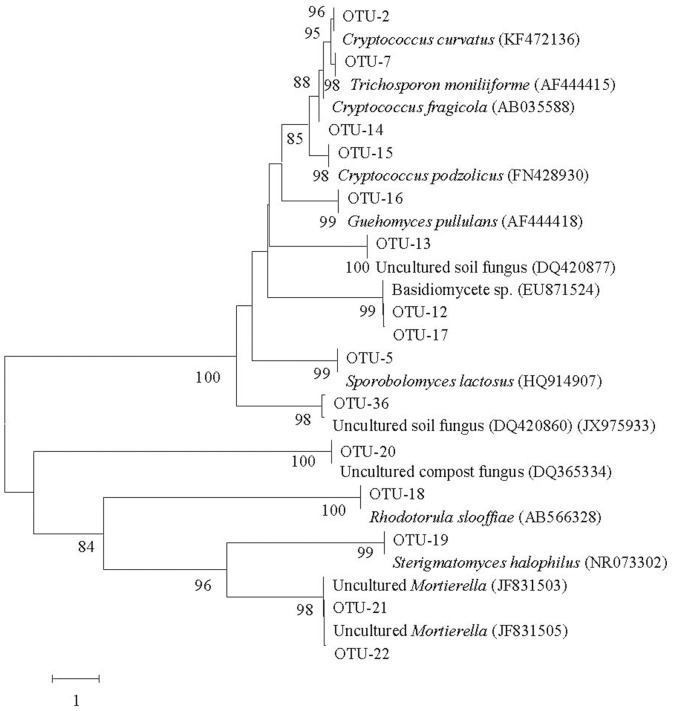
Neighbor-joining phylogenetic tree from analysis of ITS rDNA from 26 Basidomycota and Zygomycota representative sequences (OTU-21 and 22) in five libraries. The numbers at the nodes are percentages indicating the level of bootstrap support based on a neighbor-joining analysis of 1000 resampled data sets. Only values >50% are shown. The scale bar represents 1 substitution per nucleotide position.

Most of the ITS sequences from the 45 OTUs demonstrated ≥98% similarity with sequences from their closest relative taxa in GenBank. Three new sequence types, however, only demonstrated 89%–97% similarity with the existing database. Therefore, these results from the phylogenetic analysis suggest that OTU-27, 33 and 36 are novel fungal taxa that are not closely related to previous identified fungal ITS sequences in public databases (Table 2). The composition indicates that a majority (419/445) of these amplified ITS sequences belong to the phyla Ascomycota (276 clones from 29 OTUs) and Basidomycota (143 clones from 24 OTUs) (Fig. 1 and 2). The other remaining sequences belong to the phylum Zygomycota (26 clones from OUT-21 and 22) (Fig. 2).

Furthermore, the phylogenetic analyses revealed that 240 clones from 17 OTUs were most closely related to cultivable yeast forms, including three species of genus *Cryptococcus* (63 clones), *Galactomyces candidum* (52 clones), *Sterigmatomyces halophilus* (37 clones), four species of genus *Candida* (34 clones), *Trichosporon moniliiforme* (27 clones), *Rhodotorula slooffiae* (14 clones), *Basidiomycete* sp. (4 clones), *Dipodascus australiensis* (2 clones), *Guehomyces pullulans* (2 clones), *Hortaea werneckii* (2 clones) and *Sporobolomyces lactosus* (one clone) (Fig. 1 and Table 2). Another 120 clones from 20 OTUs were closely related to filamentous fungi, including *Aspergillus*, *Alternaria*, *Cladophialophora*, *Cladosporium*, *Eurotium*, *Fusarium*, *Geomyces*, *Hypocrea*, *Leptosphaeria*, *Mortierella*, *Phoma*, *Rhizoscyphus*, *Trichoderma* and *Xeromyces* (Fig. 1 and Table 2). The remaining 85 clones from 8 OTUs were closely related to uncultured fungi from soil or plant ecological systems that have been deposited in the NCBI database [37]–[41].

### Culturable fungal isolates and species richness

**Figure 3:**
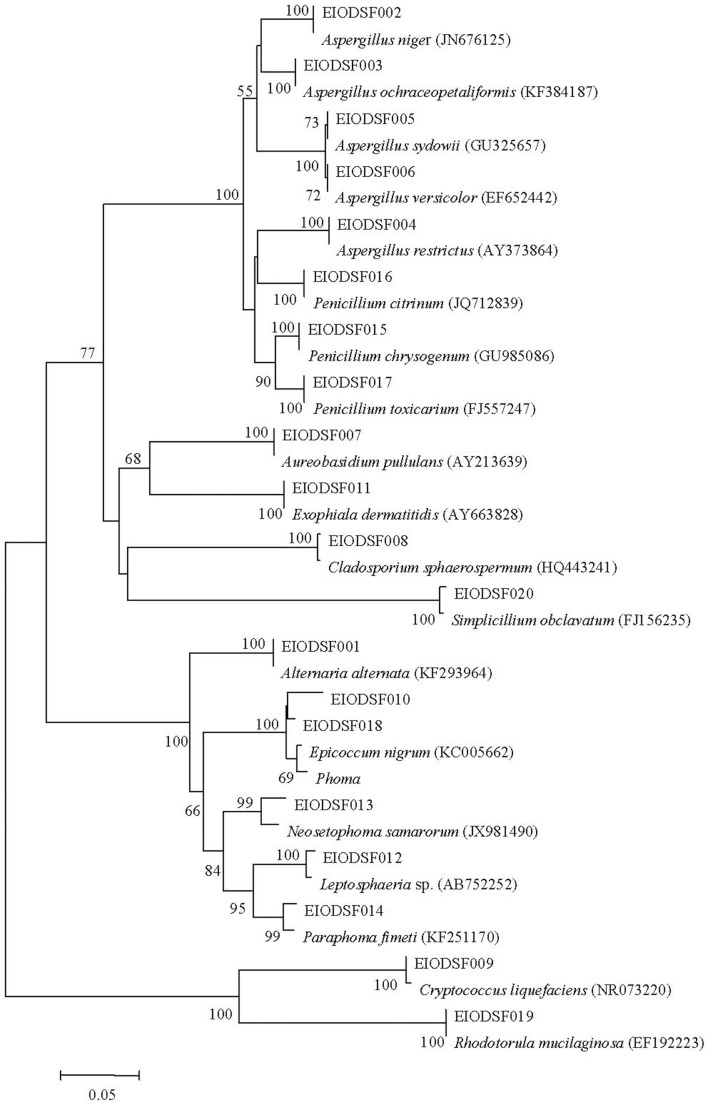
Neighbor-joining phylogenetic tree from analysis of ITS sequences from fungi isolated from five deep-sea sediments from the East Indian Ocean. The numbers at the nodes are the percentages indicating the level of bootstrap support based on a neighbor-joining analysis of 1000 resampled data sets. Only values >50% are shown. The scale bar represents 0.05 substitutions per nucleotide position.

A total of 78 fungal isolates belonging to 20 phylotypes (Fig. 3) were recovered using traditional cultivation. The ITS sequencing results showed that most of these fungal sequences demonstrated >97% similarity with sequences from their closet relative species. The one isolate EIODSF013 (accession number KJ173536), however, only demonstrates 95% similarity with the existing sequence (accession number JX981490) in the NCBI database (Table 3). A multigene analysis combined with detailed morphological and ultra structural studies, however, are needed to determine the novelty of this isolate. Most of the 78 fungal isolates belonged to Ascomycota, including two yeast and 16 filamentous fungal species. Among these species, *Aspergillus* sp., *Penicillium* sp. and *Simplicillium obclavatum* were the most diverse and common, while *Alternaria alternata*, *Aureobasidium pullulans*, *Cryptococcus liquefaciens*, *Exophiala dermatitidis*, *Epicoccum nigrum* and *Neosetophoma samarorum* were the rarest fungal species with only single or double isolates. The remaining species occurred as several isolates.

**Table 3:** Phylogenetic affiliations of culturable fungi obtained from deep-sea sediment samples A–E.

Isolates	Closest identified relative			The number of isolates				
no.	Fungal genera or species (Phylum)	GenBank accession no.	Similarity %	A	B	C	D	E
*EIODSF 001a*	*Alternaria alternata* (*Ascomycota*)	KF293964	99%	2				
*EIODSF 002*	*Aspergillus niger* (*Ascomycota*)	JN676125	99%				4	
*EIODSF 003a*	*A. ochraceopetaliformis* (*Ascomycota*)	KE384187	99%	1	1		4	1
*EIODSF 004*	*A. restrictus* (*Ascomycota*)	JX156352	99%	2	1			
*EIODSF 005*	*A. sydowii* (*Ascomycota*)	GU325657	99%	2				
*EIODSF 006*	*A. versicolor* (*Ascomycota*)	EF652442	99%	2			1	2
**EIODSF 007**	*Aureobasidium pullulans* (*Ascomycota*)	AY213639	99%					2
*EIODSF 008*	*Cladosporium sphaerospermum* (*Ascomycota*)	HQ443241	99%	2	1			3
**EIODSF 009**	*Cryptococcus liquefaciens* (*Basidiomycota*)	AF145331	99%	1				
*EIODSF 010*	*Epicoccum nigrum* (*Ascomycota*)	KC005662	98%					1
**EIODSF 011**	*Exophiala dermatitidis* (*Ascomycota*)	AY663828	99%				1	
*EIODSF 012*	*Leptosphaeria sp.* (*Ascomycota*)	AB752252	99%					6
*EIODSF 013a*	*Neosetophoma samarorum* (*Ascomycota*)	JX981490	95%					1
*EIODSF 014a*	*Paraphoma fimeti* (*Ascomycota*)	KF251170	99%			2		1
*EIODSF 015*	*Penicillium chrysogenum* (*Ascomycota*)	GU985086	99%			2		
*EIODSF 016*	*P. citrinum* (*Ascomycota*)	JN624897	99%	1		4	2	
*EIODSF 017a*	*P. toxicarium* (*Ascomycota*)	FJ557247	99%	3				
*EIODSF 018*	*Phoma sp.* (*Ascomycota*)	EF120404	99%	2	1			2
**EIODSF 019**	*Rhodotorula mucilaginosa* (*Basidiomycota*)	EF192223	99%	4				
*EIODSF 020a*	*Simplicillium obclavatum* (*Ascomycota*)	FJ156235	99%	5	3	2	2	1
Total				27	7	10	14	20

Species marked by a letter (a) are new reports for deep-sea environments. Bolded and italicized isolates are included in yeasts and filamentous fungi, respectively.

## Discussion

### New insights into the fungal communities in deep-sea environments

The fungal diversity in deep-sea environments has recently gained an increasing amount of attention. Our knowledge and understanding of the true fungal diversity and the roles this diversity plays in deep-sea environments, however, is still limited. The aim of the present study was to obtain the maximum amount of fungal diversity from deep-sea sediments collected in the East Indian Ocean. A total of 45 fungal OTUs and 20 culturable fungal phylotypes were recovered in this study (Table 2 and 3), which revealed that there is a great amount of fungal diversity in the deep-sea sediments collected in the East Indian Ocean. Recently, many studies have shown that there is an increasing number of culturable fungal species and uncultured fungal clones in deep-sea sediments from the Central Indian Basin [8], [21], [42], South China Sea [16], [18] and Eastern Equatorial Pacific [43]. Few studies, however, have specifically focused on the fungal diversity in the deep-sea environments from the East Indian Ocean. This study is the first to report new insight into the fungal communities in deep-sea sediments from the East Indian Ocean using a combination of targeted environmental sequencing and cultivation. These findings increase our knowledge and understanding of the fungal diversity in deep-sea environments.

In this study, most of the clones (94.2%) detected by targeted environmental sequencing and all of the culturable fungal isolates recovered using traditional cultivation belonged to the phyla Ascomycota and Basidiomycota (Table 2 and 3). The remaining 5.8% clones (OTU-21 and 22) belonged to the phylum Zygomycota (Table 2). In addition to these three phyla, the phylum Chytridiomycota has also been detected in many deep-sea environments, e.g. Izu-Ogasawara Trench [6]. In this study, however, no putative Chytridiomycota sequences were detected in any of the five sediment samples collected from the East Indian Ocean. We do not believe that our methods were incapable of detecting these higher taxonomic groups because the same primer set has been shown to be able to detect a diverse range of these taxa from deep-sea environments [16], [44], [45]. Therefore, these results suggest that the fungal communities in the deep-sea sediments from the East Indian Ocean tend to be dominated by Ascomycota and Basidomycota, while other fungal taxonomic groups are rare or absent.

Notably, 44.4% (20 out of 45 OTUs, Table 2) fungal OTUs and 30% (6/20) culturable fungal phylotypes (Table 3) identified in this study are new reports for deep-sea sediments (Table 2 and 3). Some of these species demonstrated phylogenetic similarity to fungal species and genera known to be found in shallow-sea environments. For examples, *A. alternata* was found in tropic sea grass *Enhalus acoroides* [45]; *E. rubrum*, *H. virens* and *Leptosphaeria* sp. were found in marine mangroves [46]; *A. ochraceopetaliformis* and *C. tenuissimum* were found in marine corals and sponges [36], [47]; *C. inconspicua* may be found in crabs [48]. Other species were affiliated with culturable or uncultured fungi from soil or plant ecological systems. For examples, *R. ericae* was isolated from lodgepole pine in central British Columbia [49]; and *D. australiensis* was recovered from agricultural soil [50]; uncultured soil fungus (OTU-13) was detected in a genetically modified rice ecosystem [41]. These results may appear to be inconsistent, but previous studies have shown that while a majority of the fungal isolates from deep-sea environments were psychrotolerant, they grew more rapidly at 30°C than 5°C [42], [51]. Moreover, most of the fungi isolated from deep-sea environments are halotolerant and do not absolutely require seawater for growth [24], [32]. It is possible that there are no true indigenous fungi in deep-sea environments and that species from terrestrial environments have gradually adapted to deep-sea extreme conditions [6].

### Yeast fungi detected by targeted environmental sequencing

Our targeted environmental sequencing results showed 53.9% fungal clones were closely related to yeast fungi. Among the yeast forms in this study, most of the clones represented phylotypes relevant to the genera *Cryptococcus* and *Galactomyces*. Some of *Cryptococcus*-related phylotypes are psychrotolerant and have been found in the majority of deep subseafloor samples from North Pond, Hydrate Ridge, Peru Margin and Eastern Equatorial Pacific [43], but *Galactomyces*-related phylotypes are rarely recovered from deep-sea environments. Four OTUs were closely related to *Candida* sp., which are considered to be associated with ecosystems in anaerobic environments [26].

In addition to the previously mentioned yeast phylotypes, *Rhodotorula*, *Sterigmatomyces* and *Trichosporon* yeasts were also found to be abundant in this study. Previous studies have shown that *Rhodotorula* spp. demonstrate remarkable ubiquity based on their presence in several different habitats, such as deep-sea sediments [52]. Phylotypes related to the genera *Trichosporon* and *Sterigmatomyces* are known pathogens or parasites of marine animals, which suggests that they may also be opportunistic pathogens or parasites of deep-sea animals [47], [53]. Furthermore, OUT-12 and 17 are related to unidentified Basidiomycetious yeasts (EU871524) and were detected in a water column from the Equatorial Indian Ocean (unpublished). The remaining yeast forms were singletons or doubletons, indicating the low abundance of these clones.

### Filamentous fungi dominated the fungal community based on traditional cultivation

In this study, filamentous fungi dominated the fungal community using traditional cultivation. The five genera *Aspergillus*, *Penicillium*, *Simplicillium*, *Cladosporium* and *Phoma* were distributed in more than three sediments from the East Indian Ocean (Table 2). Members of the mycelia genera *Aspergillus* and *Penicillium* are known to be globally distributed fungal taxa. It seems doubtful that these fungal species are indigenous to deep-sea environments, and evidence of physiological adaption of these species to deep-sea environments had been reported by Raghukumar et al. [54]. Moreover, *Aspergillus* spp. were frequently detected in anaerobic marine sediments and were shown to play an important role in the denitrification process [20]. These findings suggest that these *Aspergillus* species may play a potentially versatile role for fungi in major ecological processes in the deep-sea environments. The genus *Simplicillium* was segregated from *Verticillium* and contains 10 species [55], [56]. These *Simplicillium* species occur in a broad range of ecological niches, such diseased plant tissue, soil, human nails, dog tissue and mushrooms [55], [57]. In this study, we report for the first time the presence of *Simplicillium* sp. in deep-sea sediments. *Phoma* species are known to be associated with not only land plants but also with marine plants. Previous studies have demonstrated that many terrestrial microorganisms could accumulate in deep-sea sediments [58], [59]. One terrestrial fungal genus, *Cladosporium* was isolated from three deep-sea sediments below 3000 m in this study, which indicates that sedimentation may be an important factor responsible for the accumulation of facultative marine fungi in deep-sea sediments.

In addition, only two isolates belonging to *A. alternate* were recovered using traditional cultivation, but this species was also detected by targeted environmental sequencing in this study. Therefore, *A. alternata* should be abundant in these deep-sea sediments from the East Indian Ocean. Previous studies have shown that the genera *Alternaria* was found in nearly every survey of free-living fungal communities associated with biological soil crusts [60], [61]. Few *Alternaria* sp., however, were detected in deep-sea environments.

### Comparison of fungal community by targeted environmental sequencing and traditional cultivation

A distinct difference in the fungal community based on targeted environmental sequencing compared with traditional cultivation was that the Zygomycota spp. was not recovered by traditional cultivation but was detected by targeted environmental sequencing. Previous studies have also shown that Zygomycota spp. could be found in different deep-sea environments using targeted environmental sequencing [17], [24]. Currently, however, there are no reports of isolating Zygomycota species from deep-sea environment cultures. After a further comparison of the fungal phylotypes recovered using these two methods, it was found that the majority of the fungal phylotypes recovered using targeted environmental sequencing could not be recovered using a traditional cultivation method. This finding is consistent with the findings published by Le Calvez et al. [24], who reported that there are striking differences in the deep-sea fungal diversity results when using targeted environmental sequencing compared with traditional cultivation. These findings suggest that a combination of targeted environmental sequencing and traditional cultivation will generate a more accurate assessment of the fungal diversity in deep-sea environments compared with using targeted environmental sequencing or traditional cultivation alone. Furthermore, to obtain an even greater abundance of deep-sea fungi, it is necessary to combine the methods used in this study with other methods, such as the microscopic observation of samples appropriately staining, FISH, measuring ergosterol, metagenomic methods, and other new powerful tools expected to be developed in the future [62].

## Supporting Information

File S1 **Contains Fig. S1 Map of the East Indian Ocean, location and depth of the sampling site and Fig. S2 Rarefaction curves constructed for ITS clone libraries from each of the five sampling sites.** ITS, internal transcribed spacer.
(DOCX)
