# Supplementary material for: Insights into Deep-Sea Sediment Fungal Communities from the East Indian Ocean Using Targeted Environmental Sequencing Combined with Traditional Cultivation
Source: PLoS One. 2014 Oct 1;9(10):e109118. doi: 10.1371/journal.pone.0109118 (PMC4182876; doi:10.1371/journal.pone.0109118)
Supplement: File S1 — Contains Fig. S1 Map of the East Indian Ocean, location and depth of the sampling site and Fig. S2 Rarefaction curves constructed for ITS clone libraries from each of the five sampling sites. ITS, internal transcribed spacer. (DOCX) [file pone.0109118.s001.docx]

**Supporting information**

Insights into deep-sea sediment fungal communities from the East Indian Ocean using targeted environmental sequencing combined with traditional cultivation

Xiao-yong Zhang, Gui-ling Tang, Xin-ya Xu, Xu-hua Nong, Shu-hua Qi^*^

(**Key Laboratory of Tropical Marine Bio-resources and Ecology**/RNAM Center for Marine Microbiology/Guangdong Key Laboratory of Marine Material Medical, South China sea Institute of Oceanology, Chinese academy of sciences, Guangzhou, China)

*Corresponding author, Tel.: +86-20-89022112; Fax: +86-20-84458964

E-mail: [shuhuaqi@scsio.ac.cn](mailto:shuhuaqi@scsio.ac.cn)

Fig. s1 Map of the East Indian Ocean, location and depth of the sampling site


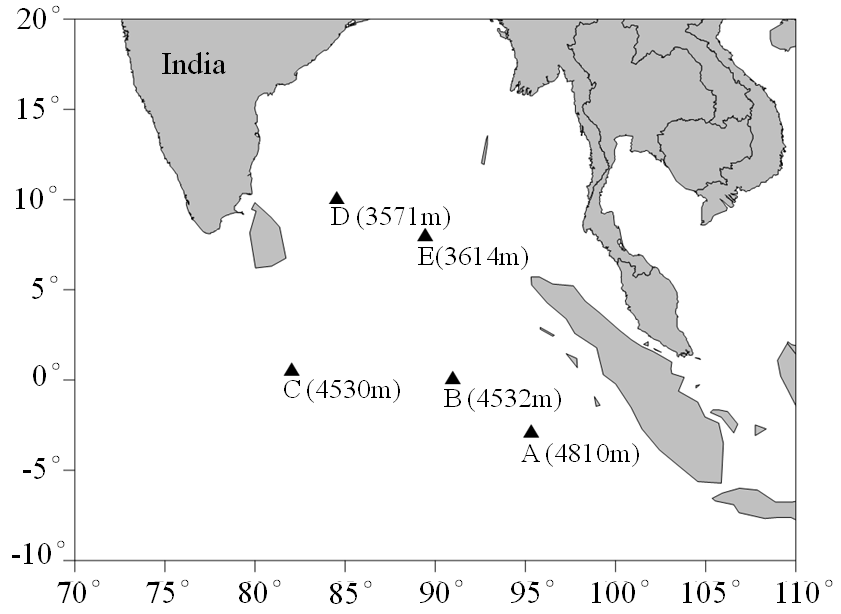

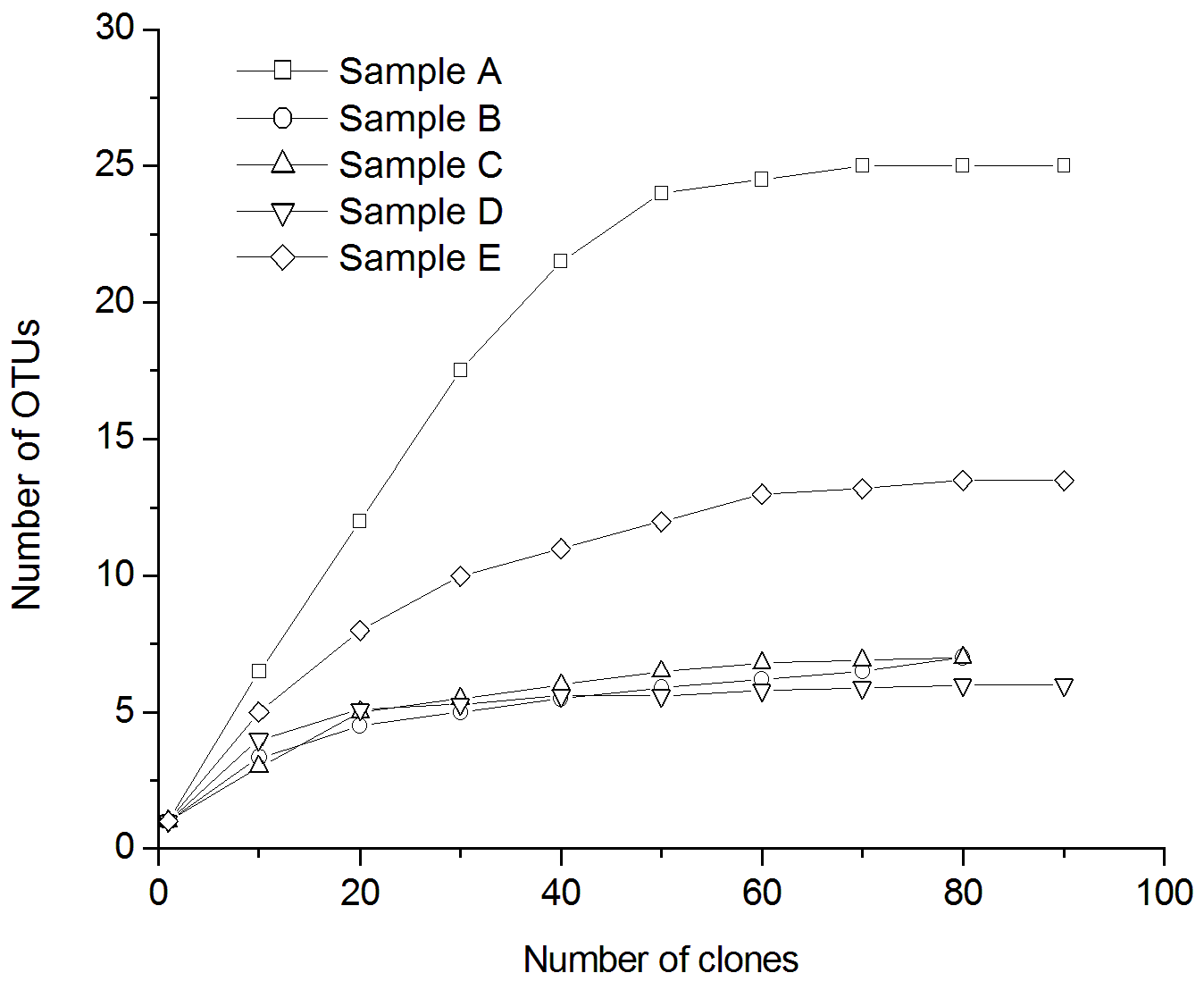


Fig. s2 Rarefaction curves constructed for ITS clone libraries from each of the five sampling sites. ITS, internal transcribed spacer
